# TD-DAQ: A low-cost data acquisition system monitoring the unsaturated pore pressure regime in tailings dams

**DOI:** 10.1016/j.ohx.2021.e00221

**Published:** 2021-08-13

**Authors:** Jack Adriaan Basson, André Broekman, Schalk Willem Jacobsz

**Affiliations:** Department of Civil Engineering, University of Pretoria, Pretoria, South Africa

**Keywords:** Geotechnical instrumentation, Soil mechanics, Waste management, Platinum tailings, Soil water retention curve, Unsaturated pore pressure regime, Tensiometer, Arduino, Pycom, SigFox, Civiltronics, IoT

## Abstract

Tailings dams are large, often self-contained, storage facilities of mine residue. On self-contained tailings dams the tailings material itself is used to raise the containment embankments holding newly deposited residue. To develop the necessary strength, it is essential that material must dry out sufficiently. Despite substantial advancements in the field of instrumentation, these parameters are rarely measured on tailings dams and their evolution over time is poorly understood. Understanding the role of pore water suction and water content evolution over time can benefit from the installation of sensors and data acquisition systems (DAQ) capable of continuously monitoring these parameters. Such monitoring remains difficult and expensive owing to the challenges of measuring negative water pressures and the often-remote locations and harsh operating environments typical of mining operations. This paper describes the development, testing and validation of a low-cost DAQ for the measurement of the unsaturated pore pressure regime in a platinum tailings dam located in the Limpopo province of South Africa. The Tailings Dam DAQ (referred to as TD-DAQ) is designed to measure the negative pore pressure, moisture content and temperature in fine-grained material over extended periods of time. These measurements are stored on the DAQ and transmitted in parallel using new wireless network communications technologies (Sigfox) suited to remote, battery powered applications. The successful deployment of the TD-DAQ presents a real-time, low-cost instrumentation solution to improve the efficiency of condition monitoring of tailings storage facilities, contributing to a reduction in the probability of failure events.

## Highlights

• Successful realisation of a low-cost data acquisition system for geotechnical applications.
• Negative pore pressure, soil temperature and dielectric permittivity measurements.
• Integrated battery power and SigFox communications for remote operation.
• High level of customizability for adding additional sensors.

## Graphical abstract

**Figure:**
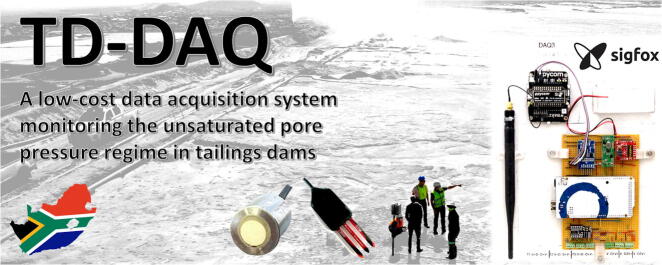

**Specifications table:** 

Hardware name	TD-DAQ (Tailings Dam DAQ)
Subject area	● Engineering and Material Science
Hardware type	● Measuring physical properties and in-lab sensors ● Field measurements and sensors
Open Source License	Creative Commons Attribution-ShareAlike
Cost of Hardware	$ 337.95 per TD-DAQ (excluding sensors)$ 946.14 per sensor configuration per TD-DAQ
Source File Repository	https://doi.org/10.17605/OSF.IO/H9VBE

## 1 Hardware in context

Tailings dam are large, often self-contained storage facilities of mine residue. Processed ore, comprising a mixture of finely ground rock and water in a slurry or paste form, is pumped from the mineral processing facility and deposited onto the tailings dam. Deposition is staggered along the footprint of the dam to allow drying of the preceding layer before new deposition occurs [1]. These large-scale structures are often at risk of failure, which poses severe environmental and economic consequences, as well as a potential loss of life should the facility not be managed and monitored correctly. Renewed awareness regarding the safety associated with these assets have developed because of recent failures widely reported in the media, such as the Brumadinho tailings dam disaster in 2019 [2]. Improved understanding of the complex unsaturated pore pressure regime in such facilities is an important aspect which can serve to reduce the risk of failure.

Optimal management of the stability of a tailings storage facility is challenging. Due to cost constraints it is desirable to keep the footprint of such facilities to a minimum. However, given a fixed mine residue production rate, a smaller footprint results in a higher rate of rise of the tailings dam. High rates of rise imply frequent deposition of new tailings, limiting the time for dry-out and strength gain and contributes to the risk of failure. The frequent deposition of wet tailings results in the development of a water table in the tailings body, with seepage typically occurring towards the outer walls. The pore water pressures below the water table are positive and are routinely measured using piezometers, most often of the standpipe type. However, above the water table pore pressures are negative and the moisture content is typically below saturation levels. Under unsaturated moisture levels, negative water pressures cause over-consolidation and strength gain of tailings during dry-out. The relationship between the moisture content and the negative water pressures is described as the soil water retention curve (SWRC). The hysteretic SWRC is the most fundamental of unsaturated soil properties and, provided that information on moisture content fluctuation with time is available, allows suction variation to be quantified. Pore water suctions play an important role in the shear strength of unsaturated tailings dam slopes.

Monitoring of suction and moisture content allow the unsaturated pore pressure regime to be tracked and interpreted in the context of the SWRC, allowing the shear strength variation of unsaturated tailings slopes over time to be better understood. Continuous condition monitoring of these dams using in-situ instrumentation provides an accurate response characterisation. However, such monitoring is not without its challenges, generally related to technological and financial constraints. The cavitation of water under negative pore water pressures means that the measurement of pore water suctions is particularly challenging. Advancements in miniaturised sensor technology, such as the development of low cost tensiometers [3], [4] now provide the ability to measure the negative in-situ pore pressures in fine grained material typically encountered in tailings dams. The cost associated with these sensors and their associated DAQ systems have hampered widespread rollout use of these monitoring systems in practice.

Modern Smart Cities - and by extension, their key infrastructure and industries, depend on a distributed, urbanised Internet of Things (IoT) to improve the allocation of public resources and value-added services for the administration, citizens and visitors [5] amid the 4th Industrial Revolution (4IR). IoT functionality is equally applicable to remote areas where condition monitoring and asset management remains challenging. Advancements in communications hardware and software have seen IoT connectivity expand beyond cellular connectivity, embracing the advantages offered by Low Power Wide Area (LPWA) radio technology and networks [6]. Two of these technologies, namely SigFox [7] and LoRa [8], respectively provide more than 98% and 99% signal transmission reliability in testing environments. The narrow-band modulation techniques employed by these technologies offer minimal noise by encoding data in a frequency band as narrow as 100 Hz [9], allowing many nodes to efficiently communicate over a much longer distance compared to other established technologies such as Wi-Fi [10]. Despite these advantages being provided at the expense of transfer rate and payload capacity, the fidelity of the range and power efficiency are ideally suited for remote structural health monitoring (SHM) applications.

This paper demonstrates the development, testing and validation of a low-cost DAQ suitable for remote, battery powered measurement of geotechnical parameters on a platinum tailings dam located in the Limpopo province of South Africa. The tailings dam DAQ (referred to as TD-DAQ) is designed to measure the negative pore pressure, temperature and moisture content in the fine-grained tailings material over a long period of time. The development of the sensor platform followed from a lack of availability of affordable off the shelf instrumentation to adequately address this monitoring project’s requirements. Deployment of the TD-DAQ compliments recent successes associated with developing customised research hardware within the Department of Civil Engineering at the University of Pretoria [11] to address an ever-diversifying array of practical applications [12], [13], [14].

## 2 Hardware description

Based on the instrumentation requirements for long-term monitoring of the pore pressure regime in tailings dams, the following design criteria were formulated:

• Primary non-volatile data storage medium with a secondary, parallel wireless data transmission system;
• Support for SDI-12 [15] and Wheatstone bridge-based sensors [16] (three of each);
• Resistance to unfavourable atmospheric and operating conditions (wind, dust, rain, UV radiation and temperature variations);
• Support for lithium polymer battery technology as the primary power delivery system with a minimum of 2 months of unattended runtime, and
• Reliable and efficient power management system.

**Fig. 1:**
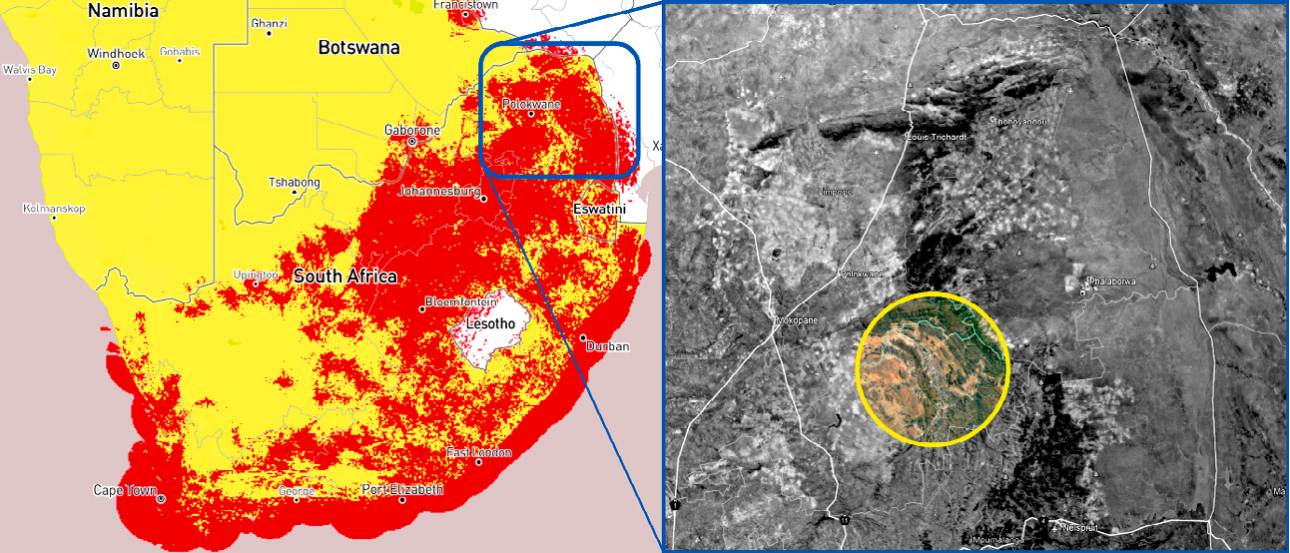
SigFox coverage (red coloured regions) for Southern Africa (as of February 2021) (left) and approximate installation location denoted by the yellow circle (right). (For interpretation of the references to colour in this figure legend, the reader is referred to the web version of this article.)

Based on the list of requirements, research was carried out to identify the most cost-effective solution based on locally available hardware platforms and capabilities. For the communications system, limited product choices were available providing for a power-efficient data transmission system. Compared to SigFox’s pre-existing coverage provided by cellular operators, the LoRaWAN (Long Range Wide Area Networks) alternative [17] requires the establishment of fixed communications infrastructure by the user [18]. Based on the available information [19], SigFox coverage (Fig. 1, left) was confirmed at the installation location highlighted by the yellow circle (Fig. 1, right). The Pycom SiPy microcontroller [20] was selected for its integrated SigFox radio module and the support for a SD card when paired with the corresponding expansion board [21]. The ubiquitous availability of general-purpose libraries to interface nearly any sensor using the Arduino family of microcontrollers was preferred to interface the platform with the required sensors and electronics. The Arduino MEGA 2560 [22] was selected for its availability, low cost, 5 V control logic and increased program memory over the Arduino Uno [23]. A dedicated serial connection between the Pycom SiPy and Arduino MEGA microcontrollers offers the advantages of a simplified interface to transmit the data between the two microcontrollers and a means to store and transmit data wirelessly using a simplified Pythonic programming environment associated with the Pycom microcontroller.

The power supply was designed to provide a regulated 5 V power source to ensure accurate measurements for Wheatstone bridge-based sensors, irrespective of the voltage supplied by the Lithium Polymer (LiPo) battery. The ability to digitally control the power regulator [24] reduces the power consumption of the sensors which are powered on only for a single measurement and data retrieval. The power delivery for all the components is controlled by a digital power switch (also referred to as the nano timer) [25] with a predefined interval between power cycles which is defined by the user. Once the data acquisition, transfer, storage and transmission cycle is complete, the control pin of the digital power switch is toggled by the Arduino microcontroller to disable power to all of the system components. This reduces the effective current draw to approximately 20nA as measured across the battery terminals. The data transmission from the SiPy is routed to the SigFox servers, as raw, unparsed data, requiring further post-processing. A callback functionality is available, allowing data to be parsed, using a simplified JavaScript program, and redirected to third party providers for data aggregation, integration and visualisation [13].

**Fig. 2:**
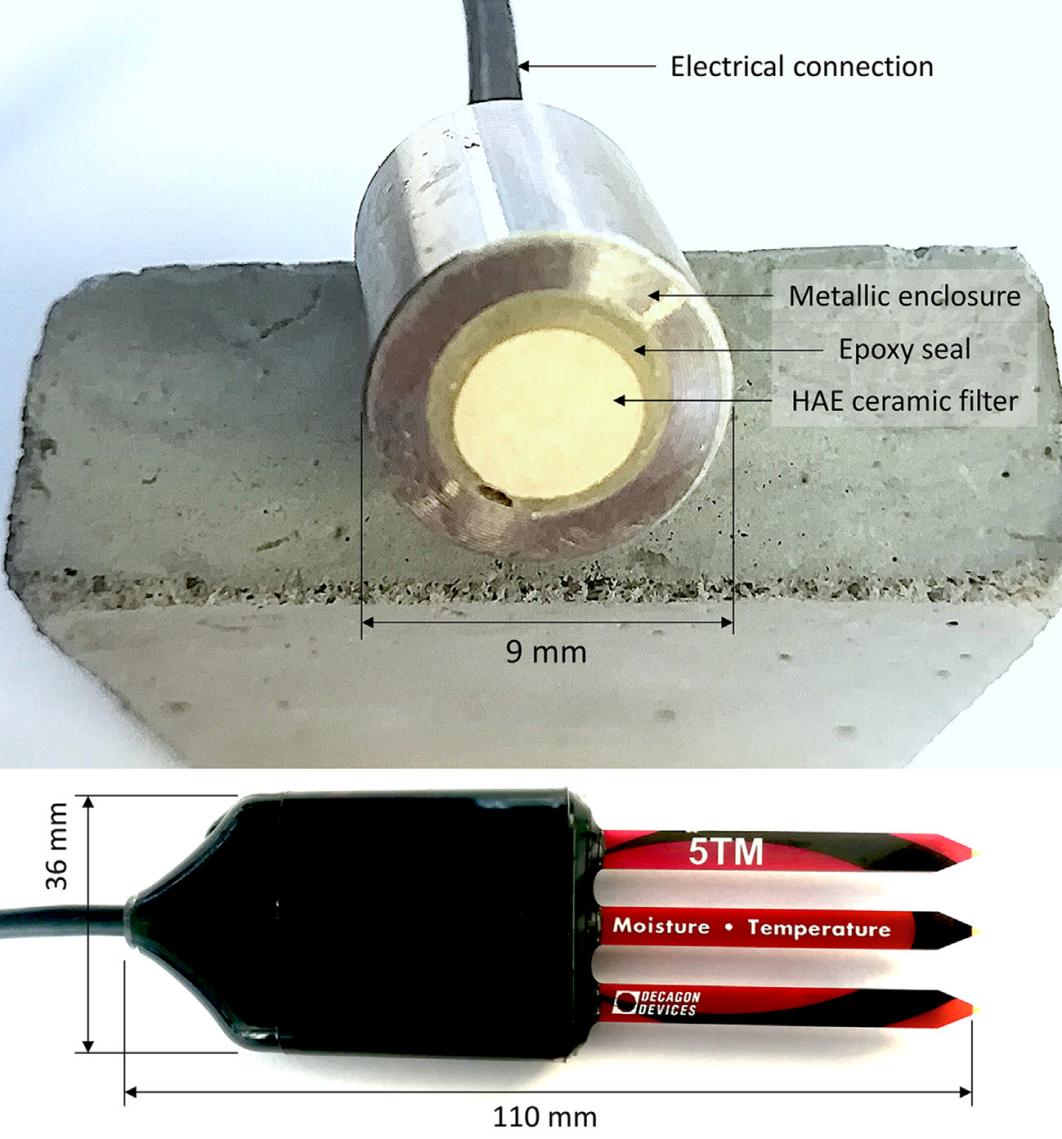
Tensiometer fabricated at the University of Pretoria (top); 5TM volumetric soil moisture and temperature sensor (bottom).

The TD-DAQ accommodates a single, 18-bit MCP3424 analogue-to-digital (ADC) converter [26] to amplify and measure the differential voltage of the tensiometers (Fig. 2, top) fabricated at the University of Pretoria. An identical ADC has successfully been used for geotechnical centrifuge testing at the University of Pretoria [14]. The 5TM [27] sensors, measuring volumetric water content (VWC) and soil temperature, are directly interfaced with the Arduino’s digital control logic (Fig. 2, bottom). The 5TM sensor design implements the SDI12 (Serial Data Interface using a 1200 baud rate) standard for communication, allowing for extended cable lengths and multiple sensors connected to the same communications bus. Accurate time information obtained from the real-time clock (RTC) sensor breakout [28] is stored alongside the data on the SD card. The coin cell battery of the RTC ensures uninterrupted operation, independent from the state of the digital power switch.

The TD-DAQ demonstrates the following advantages that may benefit the wider research community:

• General-purpose measurement of Wheatstone bridge-based instrumentation, ranging from strain gauges typically employed in structural monitoring, measurement of total soil stress using stress cells, to newer MEMS-based devices;
• Soil infiltration and interaction measurement of surface runoff;
• Estimating water balances of tailings dams, and
• Addressing a large number of devices using a shared communications bus; SDI12 sensors are typically associated with agricultural industries where remote, battery powered DAQ systems are desired to measure soil properties (moisture, temperature, salinity and acidity).

## 3 Design files

The complete list of design files is summarised in Table 1. These files provide the necessary information and firmware to duplicate and implement an equivalent TD-DAQ. These files are freely available at the Open Science Framework source file repository linked with this manuscript.

**Table 1:** Complete list of design files.

Design file name	File type	Open source license	Location of the file
Decagon_5TM.pdf	Document (PDF)	CC BY 4.0	Source file repository (Datasheets)
MS54XX Pressure Sensor.pdf	Document (PDF)	CC BY 4.0	Source file repository (Datasheets)
Arduino.zip	Arduino sketch	CC BY 4.0	Source file repository (Firmware folder)
SiPy.zip	Python script	CC BY 4.0	Source file repository (Firmware folder)
Schematic.pdf	Document (PDF)	CC BY 4.0	Source file repository (Schematic folder)
Sigfox_Decoder.xlsx	Excel	CC BY 4.0	Source file repository (Data Parsing folder)
Sensor Installation.mp4	MEGP4	CC BY 4.0	Source file repository (Installation Video folder)

*Decagon_5TM.pdf* – 5TM VWC + Temp datasheet supplied by Decagon.

*MS54XX Pressure Sensor.pdf* - Miniature SMD pressure sensor datasheet provided by Measurement Specialities™ which is used in the construction of the tensiometers.

*Arduino.zip* – Arduino sketch for the Arduino MEGA. The firmware is used to read and parse the sensor data, prior to sending the data over a serial connection to the SiPy for transmission.

*SiPy.zip* – Firmware for the SiPy module which includes both the primary source code (*main.py*) for data parsing, storage and transmission and the boot script (*boot.py*) responsible for configuring the UART port to receive data from the Arduino MEGA.

*Schematic.pdf* – Design file which provides a design overview of the electrical connections between the individual components and sensors of the TD-DAQ.

*Sigfox_Decoder.xlxs* – Excel spreadsheet used for converting the raw message payloads retrieved from the SigFox servers back to decimal format.

*Sensor Installation.mp4* – short video illustrating the installation procedure of the tensiometer sensor in the augered hole.

## 4 Bill of materials

The complete bill of materials (BOM) to replicate the TD-DAQ alongside the selection of sensors implemented (refer to the Validation and Characterisation section) is listed in Table 2 and Table 3 respectively. The listed components are not highly specialised and can be sourced from various local and international retailers.

**Table 2:** TD-DAQ Bill of Materials.

Designator	Component	Number	Cost per unit -currency	Total cost -currency	Source of materials	Material type
TD-DAQ	Arduino MEGA 2560 R3 (#MEGA-ORG)	1	$47.07 USD	$47.07 USD	Microrobotics	Other
	Pycom SiPy (#AF3534)	1	$54.56 USD	$54.56 USD	Microrobotics	Other
	Pycom Expansion Board 3.0 (#PYC-EXP3)	1	$29.10 USD	$29.10 USD	Microrobotics	Other
	Antenna (868 MHz uFL-SMA, #AF3340)	1	$7.29 USD	$7.29 USD	Microrobotics	Other
	Nano Power Timer (SparkFun TPL5110, #PRT-15353)	1	$8.20 USD	$8.20 USD	Microrobotics	Other
	ADC (DFRobot MCP3424, #DFR0316)	1	$17.58 USD	$17.58 USD	Microrobotics	Other
	RTC (Adafruit DS3231, #AF3013)	1	$16.99 USD	$16.99 USD	Microrobotics	Other
	5 V step-up voltage regulator (Pololu U3V70F5, #2891)	1	$13.35 USD	$13.35 USD	Microrobotics	Other
	Vero Board (300 mm × 180 mm, #VERO1830)	1	$4.36 USD	$4.36 USD	Microrobotics	Other
	Jumper wire (2.54 mm, variety)	1	$10.00 USD	$10.00 USD	Microrobotics	Other
	Battery (Lithium Polymer 3.7 V 5000mAh, #605068)	2	$14.13 USD	$28.26 USD	Microrobotics	Other
	Battery charger (Sparkfun, #15217)	1	$13.80 USD	$13.80 USD	Microrobotics	Other
	USB cable (2 m type C, #USB-TYPEC-2 M)	1	$2.60 USD	$2.60 USD	Microrobotics	Other
	Screw terminal (4 Pin 2.54 mm (4 Pack), #TC-4P-254)	2	$1.76 USD	$3.52 USD	Microrobotics	Other
	16 GB SD card (#SDSQUNS-016G-GN3MN)	2	$5.92 USD	$11.84 USD	Microrobotics	Other
	1.5′' Galvanised Pipe	1	$16.08 USD	$16.08 USD	RS Components	Steel
	1.5′' Galvanised pipe cap	1	$1.42 USD	$1.42 USD	RS Components	Steel
	Cable gland (Polyamide 6 mm IP68, #206–6080)	1	$7.86 USD	$7.86 USD	RS Components	Other
	3D printed mounting brackets	1	$3.00 USD	$3.00 USD	N/A	Other
Enclosure	Albro weatherproof box and brackets (350 × 250 × 200 mm IP66)	1	$41.07 USD	$41.07 USD	ARB	Composite
	Total cost per TD-DAQ	1		$337.95 USD

**Table 3:** Sensor Bill of Materials.

Designator	Component	Number	Cost per unit -currency	Total cost -currency	Source of materials	Material type
Sensors	Decagon 5TM Water content sensor	3	$281.88 USD	$845.64 USD	Campbell Scientific	Other
	Tensiometers (internal development)	3	$33.50 USD	$100.50 USD	University of Pretoria	Other
	Total cost of the sensors per TD-DAQ	1		$946.14 USD		

## 5 Build instructions

**Fig. 3:**
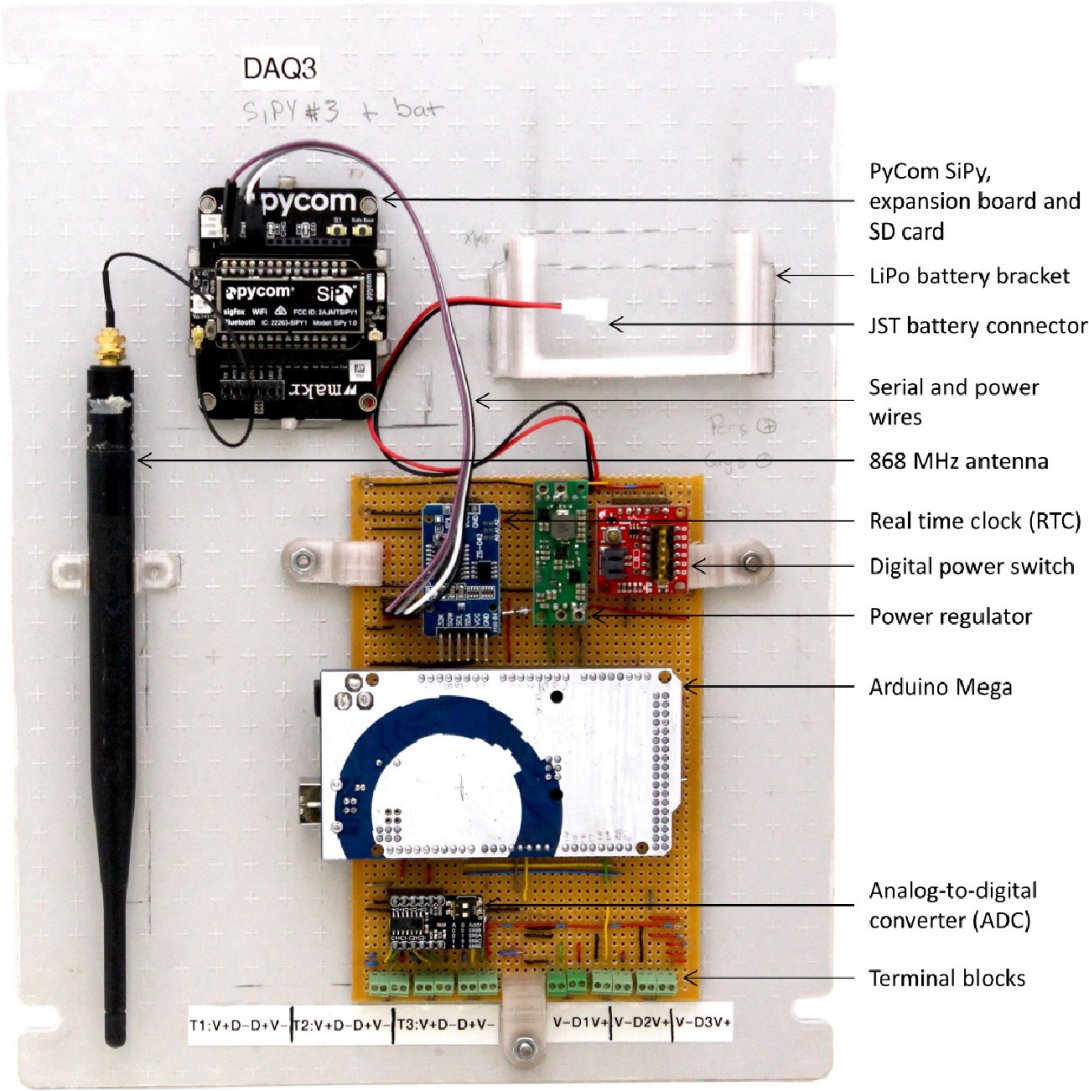
TD-DAQ electronic assembly illustrating the primary components and configuration.

**Fig. 4:**
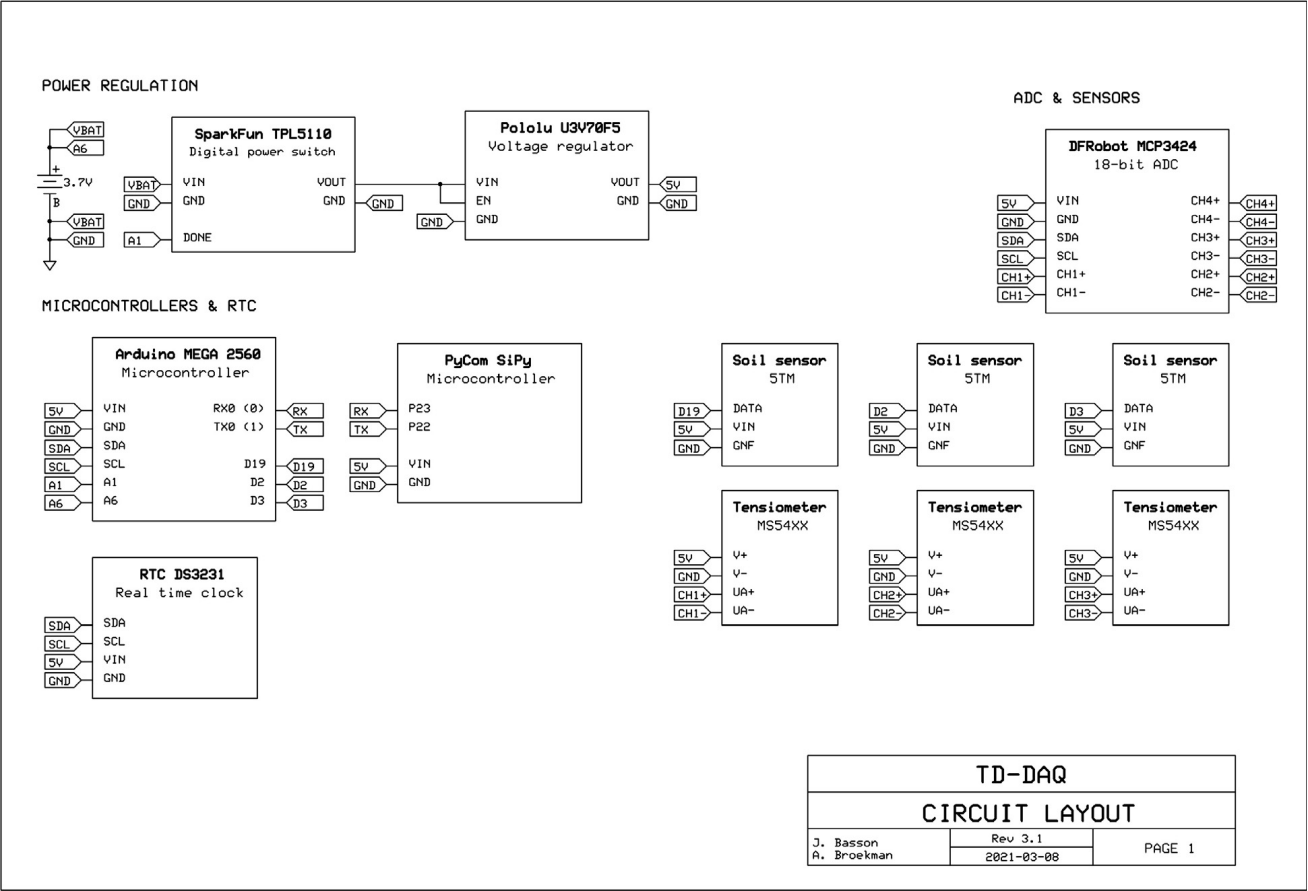
Electronic design schematic of the TD-DAQ.

Fig. 3 illustrates the primary electronics components installed onto the vero board, alongside the corresponding electronic schematic (Fig. 4) of the various components and interconnects discussed in this section. The Arduino was installed in an inverted position onto the corresponding pins, eliminating the need for additional – and often unreliable - jumper wires. The software, payload bandwidth, power supply and enclosure are discussed in separate sections.

### 5.1 Software

Software for the Arduino was developed using the standard Arduino IDE, with the Pycom SiPy software written in Atom. For the Arduino, the MCP3424 [29] and SDI12 serial [30] libraries were used to implement the ADC and SDI12 sensor interfaces respectively. The ADC features a programmable gain amplifier (PGA) which modifies reference voltage from ± 0.256 V to ± 4.096 V. Owing to the small full-scale output produced by the tensiometer’s Wheatstone-bridge design (150 mV), the smallest FS was selected (±0.256 V) to maximise the resolution efficiency (0.98 μV/step). To take advantage of the full 18-bit capabilities of the ADC, the sampling frequency was configured for 3.75 Hz, which allows all the sensors to be interrogated within one second. The SDI12 sensors features onboard circuitry which automatically converts the measurements to dielectric permittivity and temperature values. The one-wire serial data interface retrieves the information based on the unique address of each of the sensors on the bus after a specified waiting period (configured as 250 ms for the TD-DAQ implementation). To avoid the need for specific addresses and potentially incorrect wiring of the sensors during installation on site, each sensor was allocated a dedicated digital pin on the Arduino for communications. After successfully obtaining the sensor measurements, the date-time string is constructed from the RTC along with the measured battery voltage. The 5 V logic of the Arduino MEGA eliminates the need for a voltage divider typically implemented for 3.3 V architectures to measure the battery voltage.

Once a predetermined amount of time has passed which ensures the SiPy is ready to receive data over the serial interface, the Arduino transmits the combined data string (using a serial print statement) as a set of characters to the SiPy. This data is intercepted from the Arduino’s TX and RX pins which are connected to the SiPy’s corresponding serial communication pins. Once the SiPy receives the completed serial string, it in turn is parsed as a sequence of variable data associated with the sensor measurements. The data is stored on the SD card in a comma separated variable (CSV) file, followed by transmission using the integrated SigFox radio modem. This time duration of this process is also well defined with minimum deviation. As a result, 24 s after the Arduino originally transmitted the data to the SiPy, the Arduino pulls the reset pin of the digital power switch to terminate power to all the microcontrollers, electronics and sensors.

### 5.2 Payload bandwidth

Due to the inherent limitations of SigFox, a maximum of 140, 12-byte uplink messages per day can be accommodated by the module. For geotechnical applications, transient events are typically not considered, negating the need for high frequency data transmission. Intervals as short as 11 min remain within the uplink budget; the TD-DAQ is configured for an hourly power and data transmission cycle to maximise the available battery capacity. The limited bandwidth of the messages requires rescaling of the measurements over a sufficiently narrow range to retain the required resolution [31]. This scaling operation is reversed to obtain the original measurements during the post-processing phase. Representation of values as either unsigned integers (single byte, 8-bits) or unsigned floats (two bytes, 16-bits) accommodate 256 (2^8^) and 65,536 (2^16^) possible values, respectively. For the ADC measurements, the values are represented as unsigned floats, rescaled for values ranging between 0 and 150 mV (the expected maximum voltage associated with the tensiometer). The VMC and temperature measurements are represented as unsigned integers, scaled between 0 and 50 (no units) and 0 – 50 °C, respectively. The terminal voltage of the battery is measured directly by the Arduino’s internal ADC, represented as an unsigned integer over a range of 0 – 5 V. To accommodate the 12-byte payload capacity, only two of the three dielectric permittivity values are incorporated, alongside the three tensiometer measurements and the battery Voltage.

### 5.3 Power supply

The LiPo battery supplies a nominal output voltage of 3.7 V which varies from 4.15 V when fully charged, down to 3.3 V, whereupon the internal battery regulator disables the battery from over discharge. The output voltage provided by the 5 V power regulator was measured to be within 10 mV of the target value. Based on the results from extended field testing, the 5000 mAh capacity of the battery is sufficient for three months of continuous service before requiring a recharge or replacement with a fully charged battery.

### 5.4 Enclosure

The enclosure is provided with a plastic plate which is secured to the back of the enclosure. All the electronic components and brackets (fabricated using a 3D printer and PETG filament) are secured to the plate prior to installation. Terminal screw blocks ensure rigid and secure connections for the sensor wires accommodated using cable glands installed on the bottom of the enclosure to prevent the ingress of moisture. The enclosure includes a key lock for added security, tamper proofing, as well as providing ease of access to periodically replace the SD card and battery. Instructions were attached to the inside of the enclosure for the site engineers tasked with the periodic exchange of the SD cards and batteries.

## 6 Operation instructions

Prior to installation of the sensors, a calibration procedure was followed to ensure the accuracy and precision of the tensiometer measurements over the range of expected pressure values which would be encountered in the field.

### 6.1 Initial setup and calibration

**Fig. 5:**
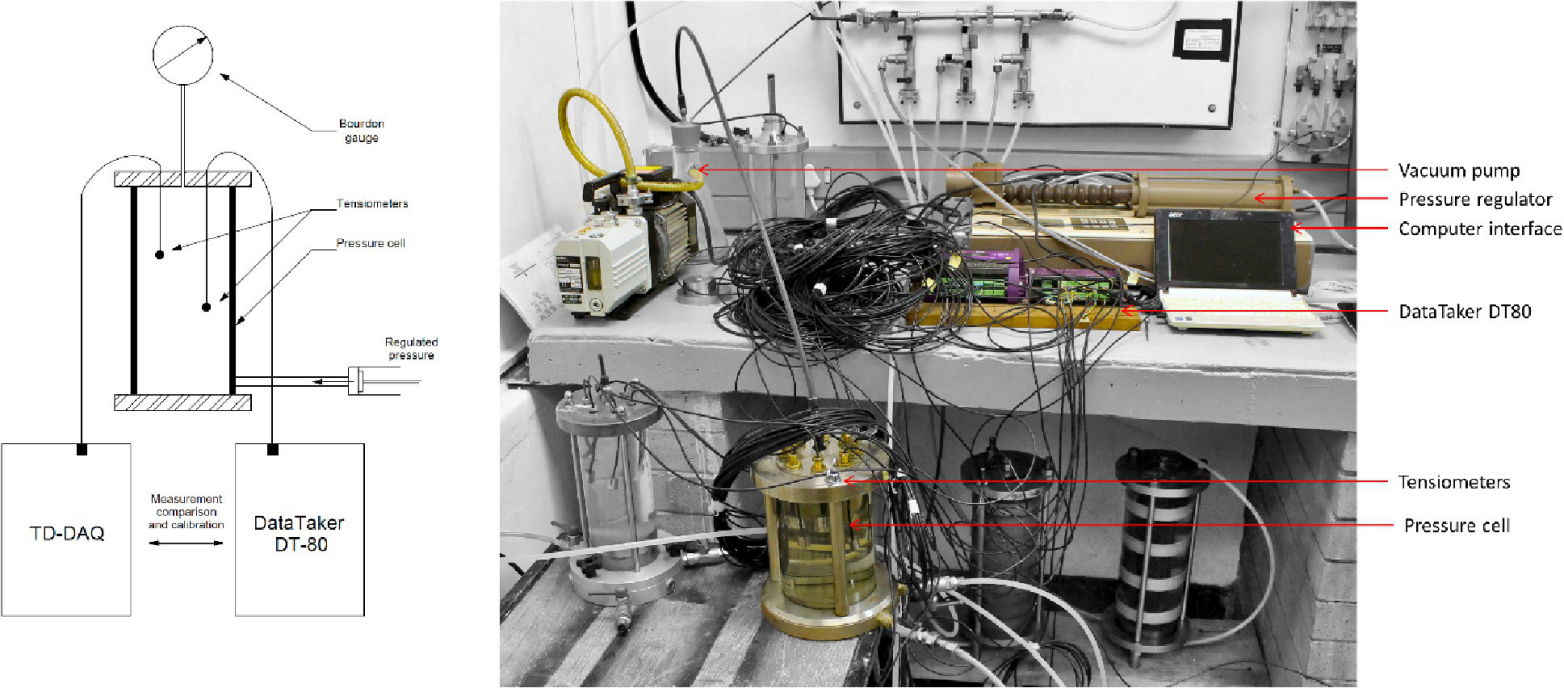
Calibration configuration schematic (left) and corresponding implementation in the geotechnical laboratory (right).

**Fig. 6:**
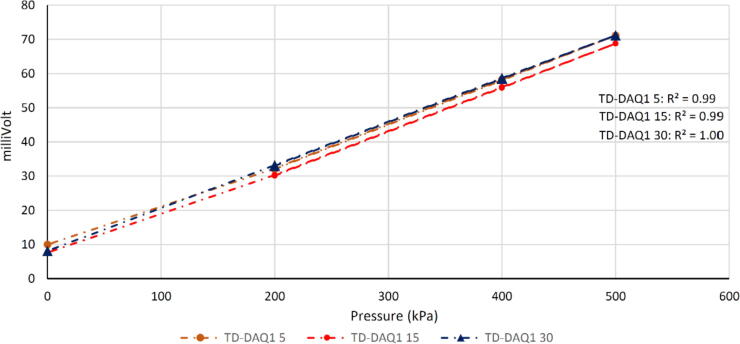
Calibration curves of the tensioners measured by TD-DAQ 1.

The calibration procedure entailed the installation of the saturated tensiometers inside of a sealed pressure cell and comparing the differential voltage measurements between those recorded by the TD-DAQ and a commercial dataTaker Series 4 DT80 [32] data acquisition system (Fig. 5). The digital power switch (Fig. 3) was configured to increase the data acquisition frequency to approximately 1 min to accelerate the calibration process. This verification step ensures that the proposed alternative (TD-DAQ) provides the required performance compared to commercial products (dataTaker Series 4 DT80). The 5TM sensors were not calibrated within a specific soil medium as the Topp equation [33] is used to convert the dielectric permittivity to VMC. The calibration cell pressure was adjusted to 0, 200, 400 and 500 kPa whilst monitoring the response of the tensiometers, allowing the differential voltage measurements to equilibrate after every adjustment (Fig. 6). This process was repeated twice; once for the sensor connected to the TD-DAQ, the other for the sensor connected to the dataTaker Series 4 DT80. Excellent agreement was found between the two configurations, despite the pressure controller being operated manually. The pressure sensor (tensiometer) provides a linear response to the change in pressure. As such, the calibration factor is determined by calculating the gradient of the pressure-differential-voltage curve. Similarly, the zero offset is equal to the differential voltage measured when no pressure is applied (0 kPa). Every tensiometer is calibrated individually to eliminate performance differences resulting from the imprecise, manual fabrication process of the sensors. The performance of the TD-DAQ is limited only by the accuracy of the tensiometers; the ADC provides a resolution of 15 Pa and a corresponding full-scale of ± 2.072 MPa. Pre-wiring minimises the risks associated with incorrect sensor wiring on site where exposure to the elements posed difficult working conditions, the limited time available for the installation and travel restrictions associated with the national Covid-19 lockdown curfew imposed at the time. The digital power switch was reconfigured after the calibration process to an interval of 1 h.

### 6.2 Sigfox data storage and retrieval

**Fig. 7:**
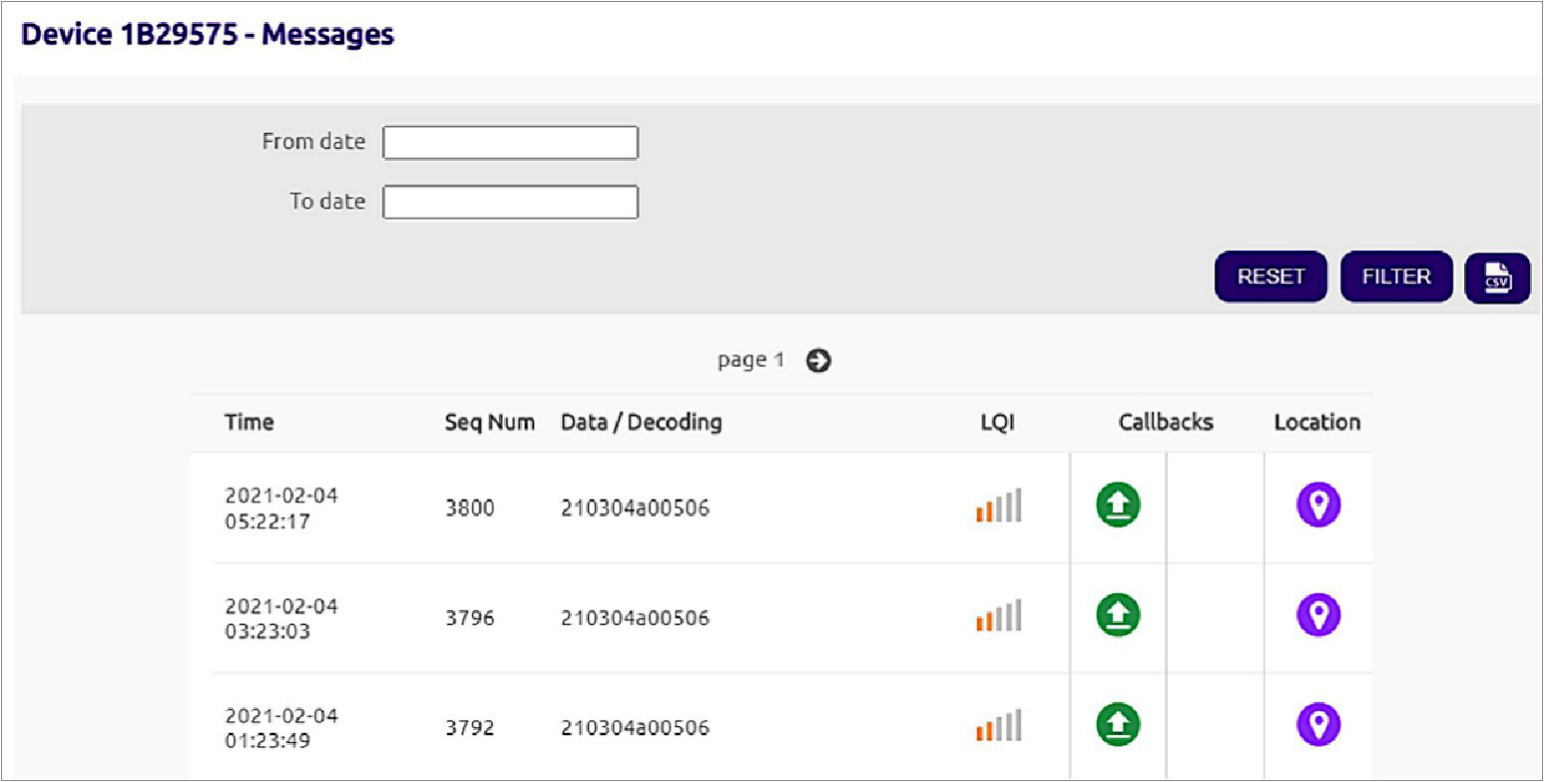
Sigfox backend illustrating the unparsed message payloads and signal strength.

**Fig. 8:**
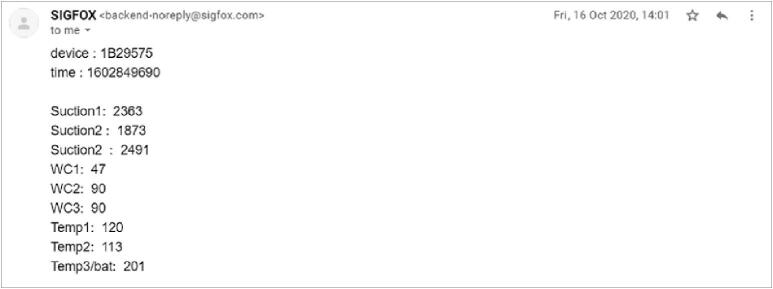
Example of the parsed Sigfox message received by e-mail service.

Data transmitted from the TD-DAQ using the SigFox network is aggregated on SigFox’s own dedicated storage servers and is accessible to the end user using authorised credentials to access the service (Fig. 7). The message payload can be configured for sending the parsed data to a third-party service or other communications services such as e-mail. This callback functionality does not incur any additional charges besides that of the connectivity fee associated with the SigFox device. An example of parsed data received using the e-email service is illustrated by Fig. 8, providing a simple method to monitor the status of the hardware. The historical data can be aggregated and downloaded as a single CSV file for postprocessing and analysis.

**Fig. 9:**
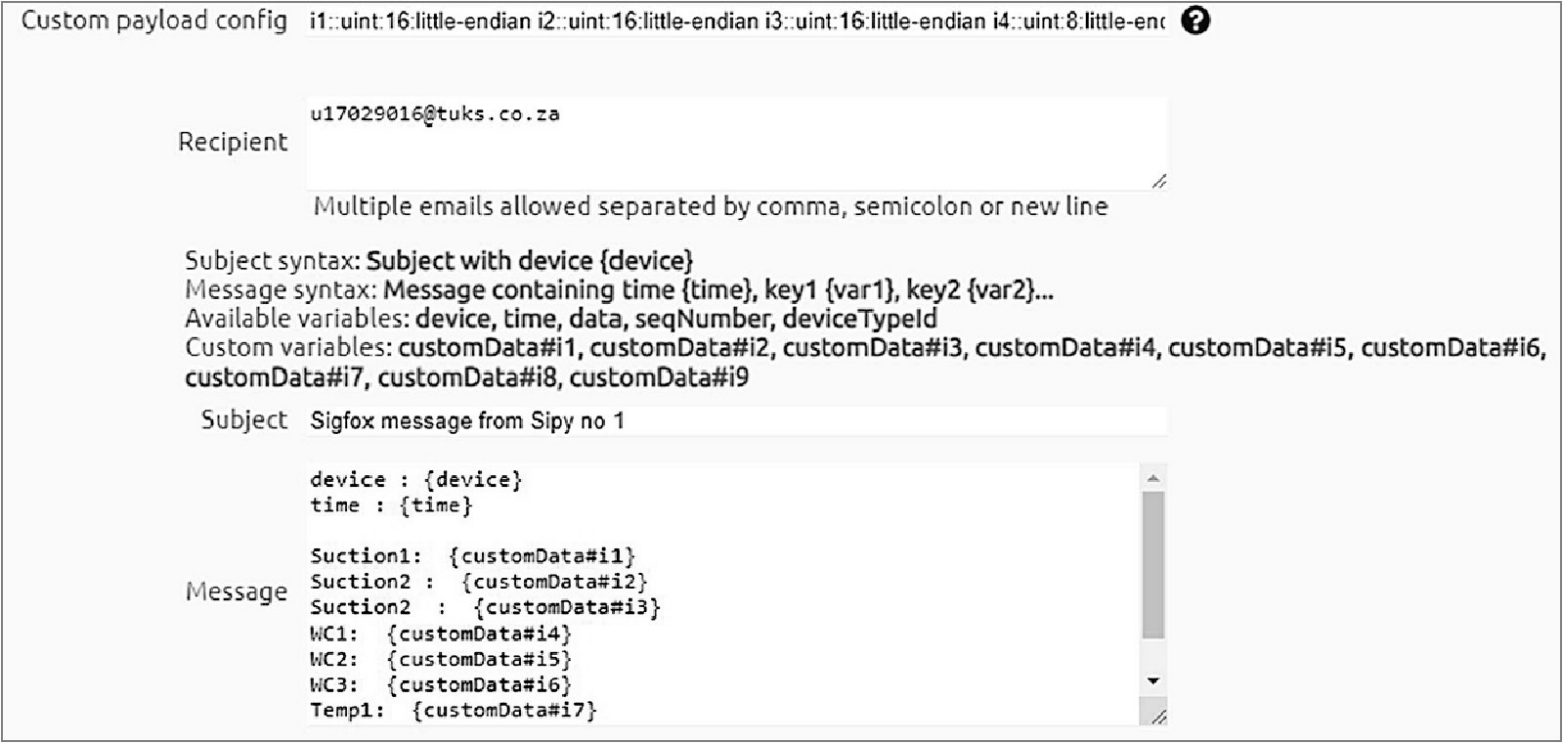
Configuration of a callback used to parse the payload messages and interface the data with a third party API.

As noted in the discussion surrounding the available payload bandwidth of each message that requires transmission over the SigFox network, a specific parsing strategy is utilised for every variable. This is reflected in the callback configuration where each variable is defined alongside the corresponding size (in bytes) and endianness. The three tensiometer values are encoded as unsigned words (16-bits), with the remaining six variables (three dielectric permittivity, two soil temperature and battery voltage) encoded as unsigned bytes (8-bits). The values are constructed as part of the e-mail message (Fig. 9) using JSON (JavaScript Object Notation) formatting. The implemented custom payload configuration:

i1::uint:16:little-endian

i2::uint:16:little-endian

i3::uint:16:little-endian

i4::uint:8:little-endian

i5::uint:8:little-endian

i6::uint:8:little-endian

i7::uint:8:little-endian

i8::uint:8:little-endian

i9::uint:8:little-endian

The implemented JSON message custom payload configuration:

device : {device} #Get device name

time : {time} #Time when message was received

Suction1: {customData#i1} #Variable corresponding to Suction1

Suction2 : {customData#i2} #Variable corresponding to Suction2

Suction3 : {customData#i3} #Variable corresponding to Suction3

WC1: {customData#i4} #Variable corresponding to WC1

WC2: {customData#i5} #Variable corresponding to WC2

WC3: {customData#i6} #Variable corresponding to WC3

Temp1: {customData#i7} #Variable corresponding to Temp1

Temp2: {customData#i8} #Variable corresponding to Temp2

Temp3/bat: {customData#i9} #Variable corresponding to Temp3/bat

For processing larger datasets downloaded as a CSV file, either a spreadsheet (available from the source file repository) or programming script can be utilised to decode the data from the hexadecimal format to individual variables in decimal format. The dataset is downloaded separately for every SigFox device, which includes the timestamp when the message payload was received by the server. Optional metadata pertaining to the approximated geolocation and signal strength is also available for every entry in the dataset. The payload configuration implements a little-endian format compared to the big-endian format provided by the CSV file; transposing the bytes (for 16-bit integers) resolves this difference. Finally, the data mapping process implemented by the Arduino script is simply reversed, yielding the original measurements with the correct units. An example of the header of one such CSV data file is illustrated below:

Data;“Timestamp”

8f07f807b2073c5429776dcd;“2020-10-12 19:13:32″

8807da07af073e5329766dcd;“2020-10-12 16:13:54″

8607d607b1073e5329766dcd;“2020-10-12 15:14:02″

8807d307b1073e5329766dcd;“2020-10-12 14:14:09″

9207d807b4073f5329756dcd;“2020-10-12 13:14:16″

## 7 Validation and characterization

**Fig. 10:**
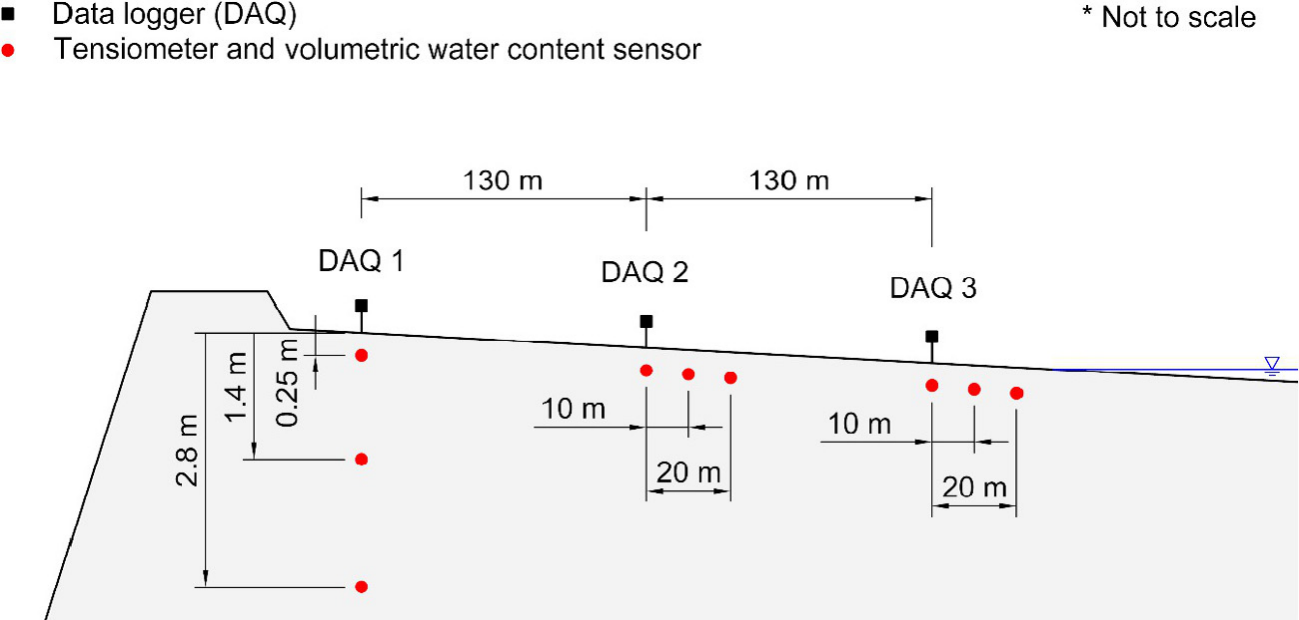
Cross section of the tailings dam illustrating the dimensions of the sensor installation.

**Fig. 11:**
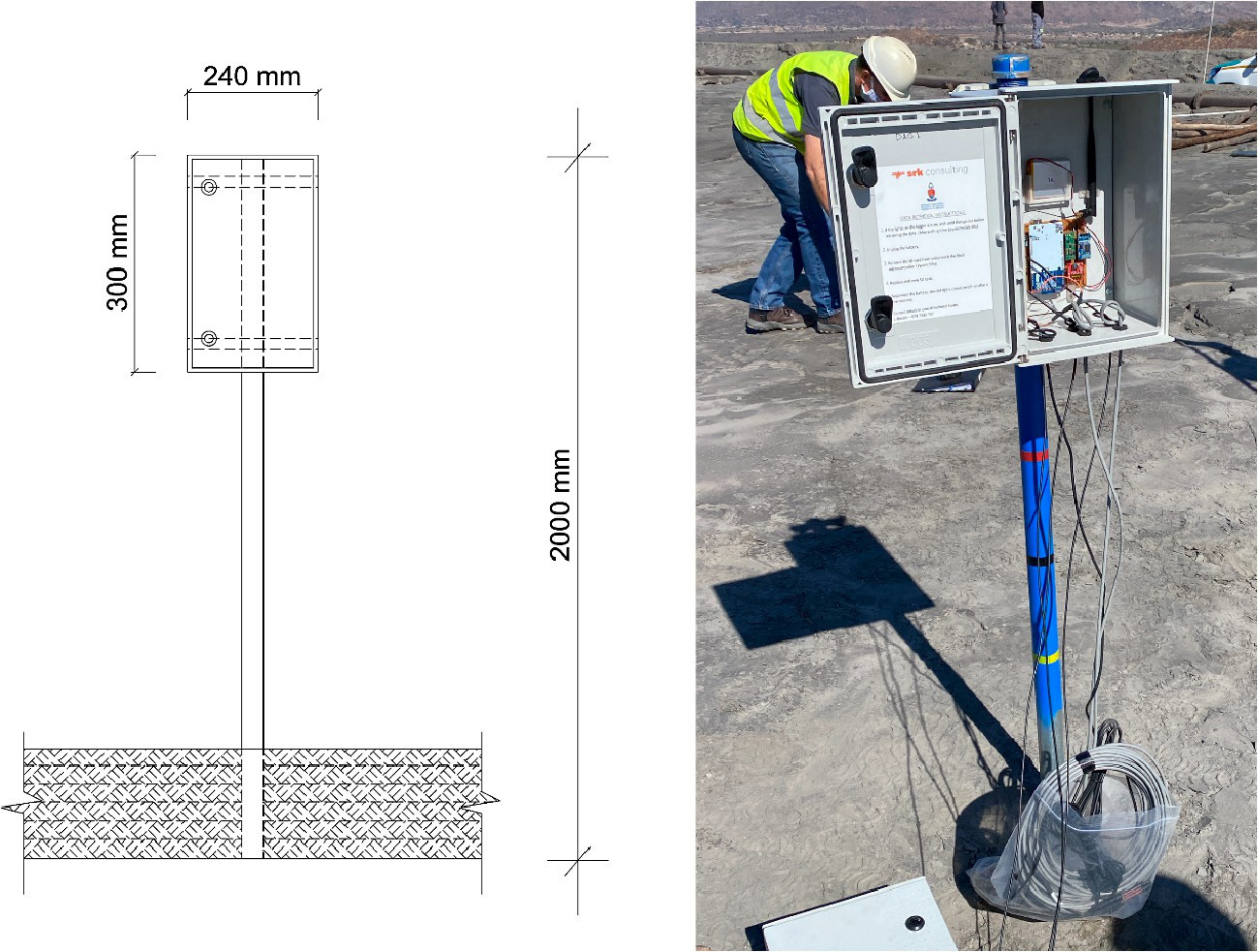
Illustration (left) and corresponding photograph (right) of the TD-DAQ enclosure installed onto the pole.

**Fig. 12:**
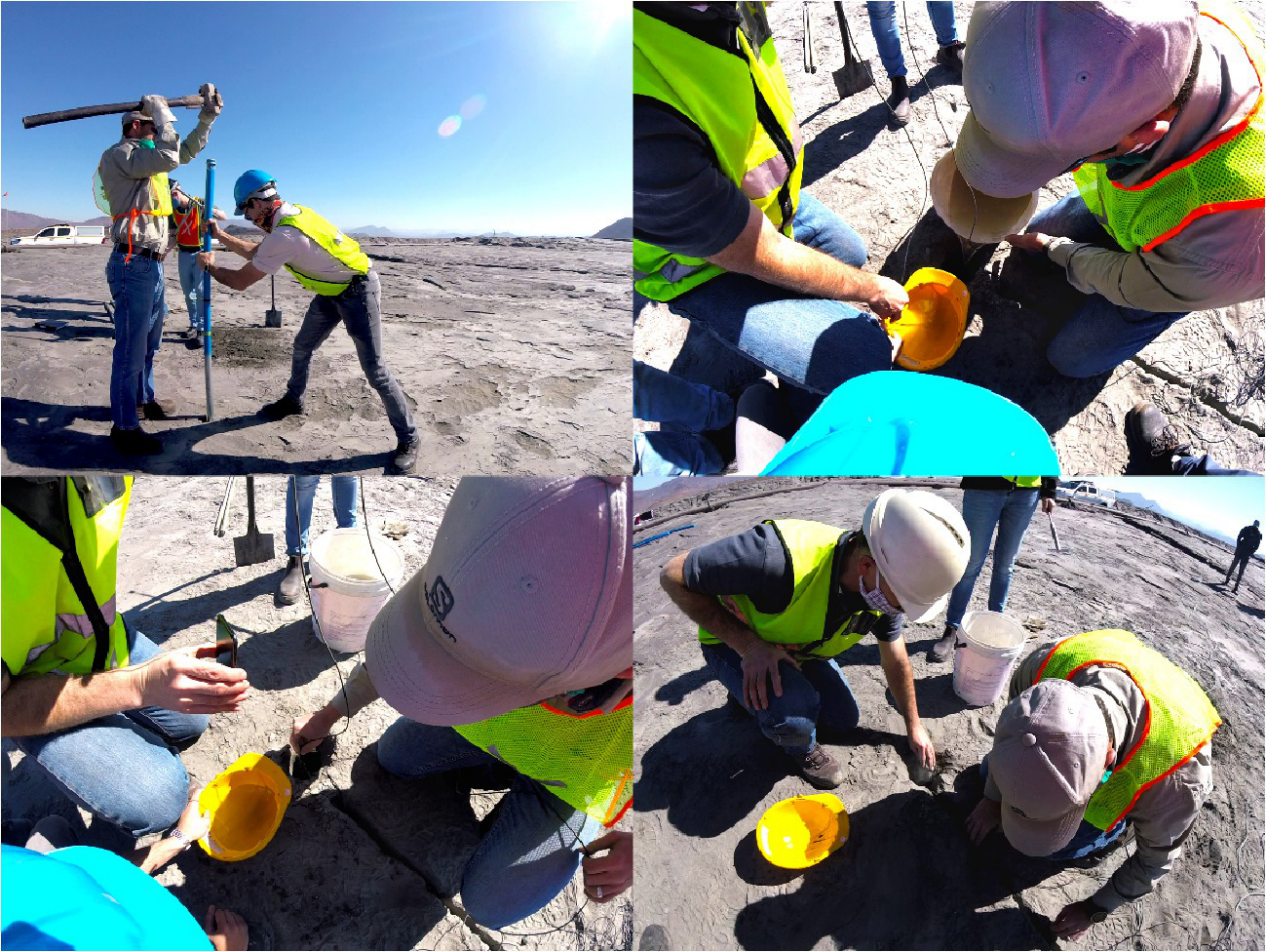
Installation methodology: pole installation (top left), tensiometer transfer from the water bottle into a smaller container (top right), installation (bottom left) and backfilling (bottom right).

The validation and characterisation consider the field installation and results obtained after an extended period of data collection. The platinum mine is situated approximately 350 km northeast Pretoria (Fig. 1, right). The mine operates a tailings dam with a footprint of 48 ha on which deposition occurs by means of spigotting. A total of three TD-DAQs were installed approximately 130 m apart from one another (Fig. 10). The TD-DAQs were attached to 75 mm diameter galvanised steel poles, driven approximately 500 mm into the tailings to allow adequate freeboard for the tailings level to rise as deposition proceeds (Fig. 11, left). During sensor installation special care was required to avoid the tensiometers drying out (Fig. 12). The sensors were kept submerged in water right up to the point that they were lowered into hand-augered holes drilled for installation. The holes were carefully backfilled with the excavated tailings and lightly compacted by hand. A short video demonstrating the tensiometer installation process can be accessed from the source file repository associated with this article. The VMC sensors were installed in a similar manner, lowered to the bottom of the borehole, slotted into the end of a PVC tube so that the sensor prongs (refer to Fig. 2) could be pushed into the material at the bottom of the hole. Redundant lengths of cable were looped and fixed to the pole, allowing the DAQ to be raised as tailings deposition progressed over time. The battery was installed immediately after sensor installation.

**Fig. 13:**
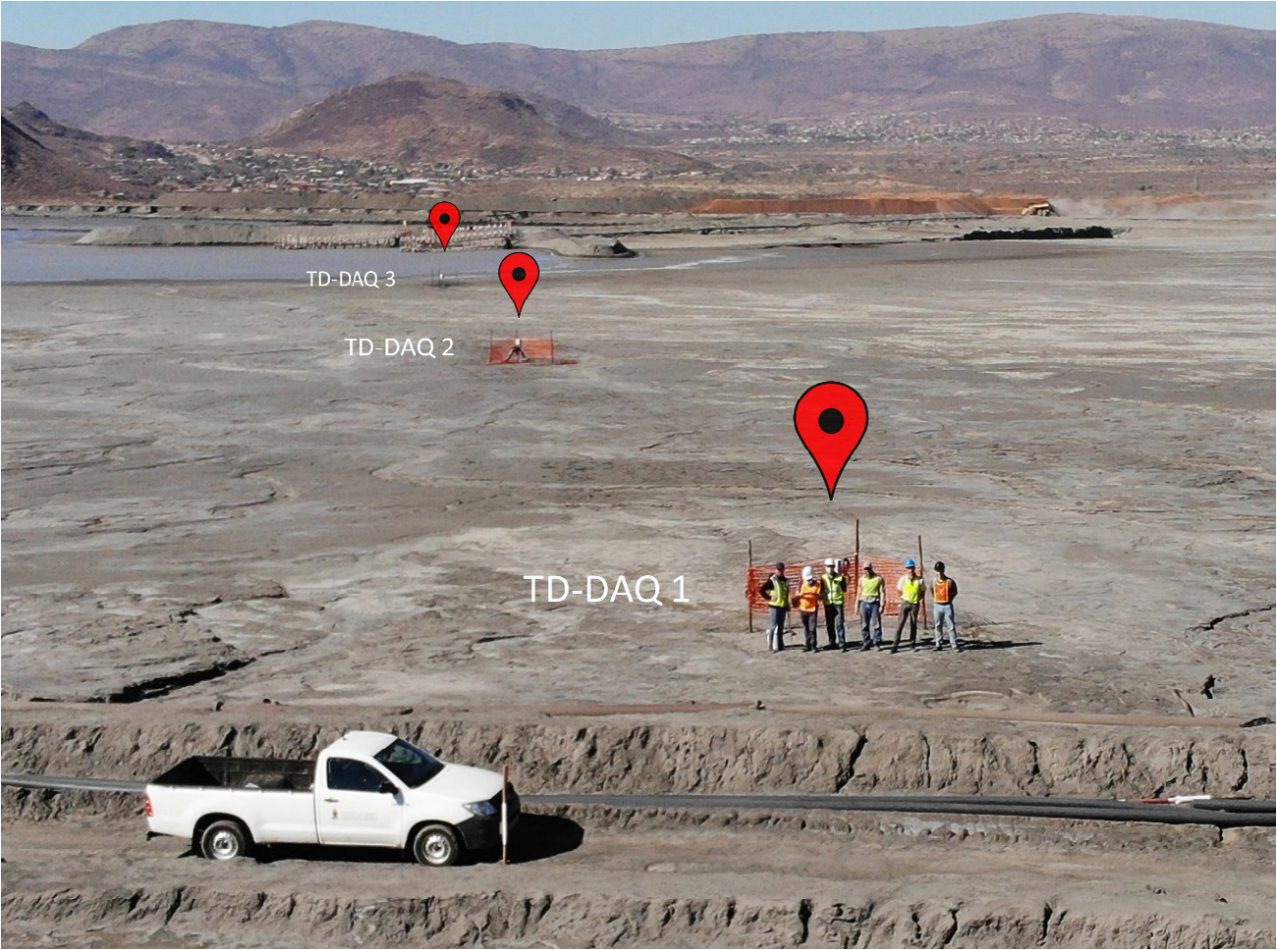
UAV photograph of the three installed TD-DAQs.

**Fig. 14:**
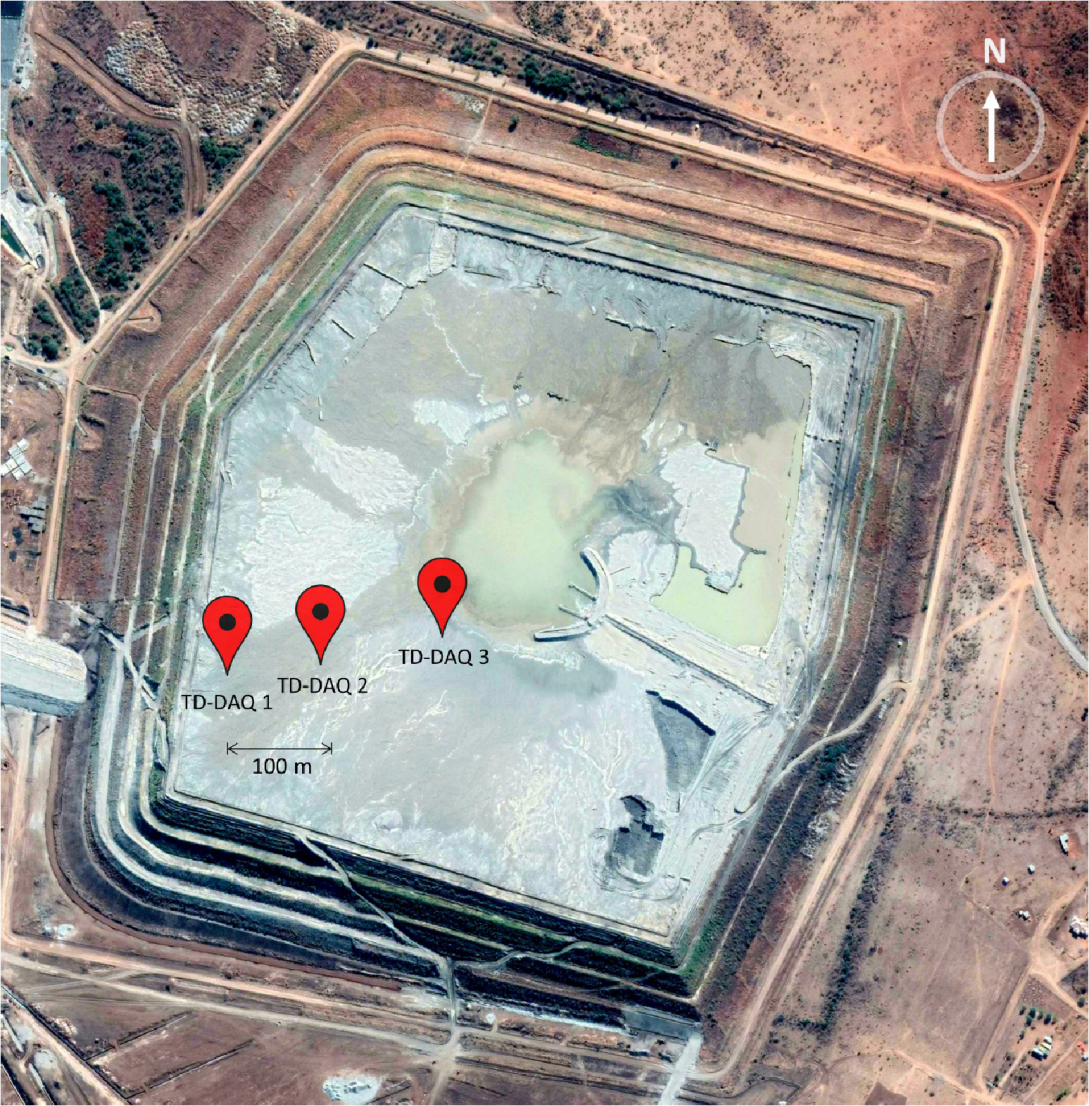
Installation location of the three TD-DAQs on the tailings dam (Google Maps).

Fig. 13 and Fig. 14 and illustrate the three TD-DAQs on the tailings dam after installation.

**Fig. 15:**
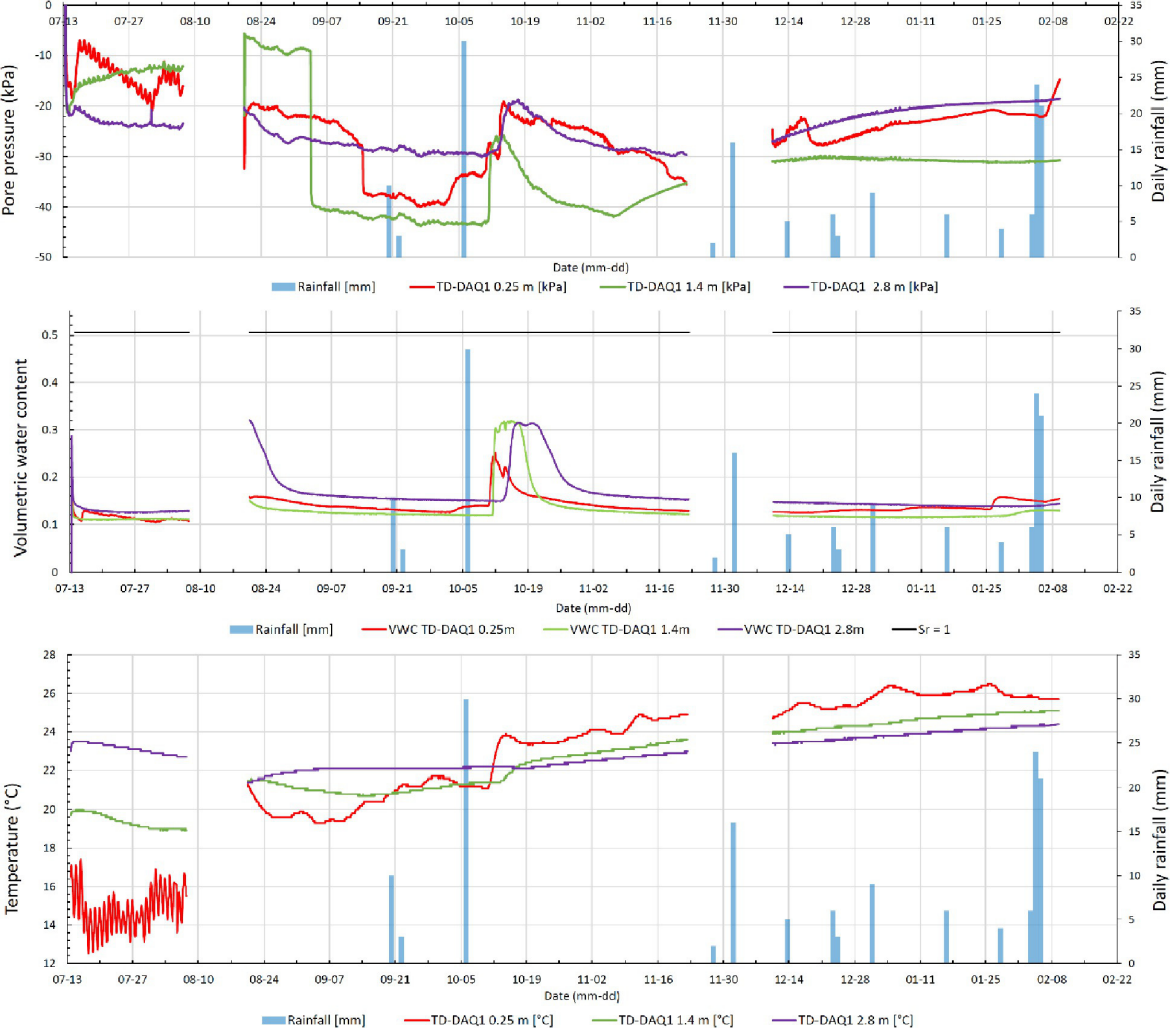
Time history of the pore pressure (top), volumetric water content (center) and temperature (bottom).

Fig. 15 presents the pore pressure, volumetric moisture content and temperature history of TD-DAQ1 from 13 July 2020 to 22 February 2021, with the rainfall data recorded at a nearby weather station. The area typically receives summer rainfall between November and March. The first month of data (July) is characteristic of daily temperature variations and increased soil suctions following the installation of the sensors. The absence of data for the month of August and November were due to the battery discharging. The long-term trend of increasing temperatures during the latter part of the year was expected, with transient events associated with rainfall and deposition events of particular interest assisting an improved understanding of changes in the unsaturated pore pressure regime in the tailings.

Based on the experience gained from the development process and field performance, the following capabilities and limitations of the TD-DAQ are summarized:

• Reliable, long-term operation was achieved by the TD-DAQ as originally designed for the particular application;
• Despite the low-cost design of the hardware, the performance ultimately delivered the quality and accuracy typically expected from commercial hardware solutions;
• The power efficiency eliminates the need for both high-capacity batteries or solar cells which are prone to theft and damage in isolated field installations. To date, the battery capacity has proven to be sufficient for at least three months;
• The relatively large number of components resulted in construction of multiple hardware units being time consuming. New generations of wireless microcontrollers should reduce the labour and time required to construct platforms of a similar specification;
• The simple serial interface between the Arduino and SiPy microcontrollers proved reliable. The SD card storage served as the primary data storage medium which was exchanged periodically to retrieve the data;
• The small, lightweight construction of the hardware was convenient to install in the field;
• The successful demonstration of SigFox as a viable communications service provides a development roadmap to deploy more reliable and integrated communications infrastructure on future projects requiring similar functionality in challenging environmental conditions;
• Unreliable SigFox coverage at the installation location resulted in intermittent data communications; despite this, periodic data transmission provided an indication of activity and battery charge for one of the loggers. LoRaWAN technology can be explored to mitigate unreliable reception through installation of dedicated gateways and to increase the number of sensors which can be accommodated using the available bandwidth, and
• During the testing and validation phase, various jumper cables had to be replaced due to intermittent, unreliable connectivity. More secure, permanent wiring assemblies are recommended to reduce the probability of malfunctioning connections between components and sensors which are difficult to troubleshoot.

## CRediT authorship contribution statement

**Jack Adriaan Basson:** Methodology, Software, Validation, Formal analysis, Investigation, Resources, Data curation, Writing – original draft, Visualization, Project administration. **André Broekman:** Conceptualization, Methodology, Software, Writing – original draft, Writing - review & editing, Visualization. **Schalk Willem Jacobsz:** Formal analysis, Investigation, Resources, Writing - review & editing, Supervision, Project administration, Funding acquisition.

## Declaration of Competing Interest

The authors declare that they have no known competing financial interests or personal relationships that could have appeared to influence the work reported in this paper.
